# Parenthood as intended: Reproductive responsibility, moral judgements and having children ‘by accident’

**DOI:** 10.1177/0038026119868643

**Published:** 2019-08-01

**Authors:** Robert Pralat

**Affiliations:** University of Cambridge, UK

**Keywords:** accident, family, intention, parenthood, pregnancy, reproduction, responsibility, sexuality

## Abstract

What does it mean to have a child ‘by accident’? And why is parenthood so often described as happening ‘accidentally’, even when it is likely to involve at least some degree of intention? Drawing on interviews conducted in England and Wales with lesbians and gay men who do not have children but may have them in the future, this article explores the meanings of the notion that, as a same-sex couple, ‘you can’t have a child by accident’ – a comment that interviewees frequently made unprompted when they were asked about the possibility of becoming parents. My data show that referring to ‘accidental parenthood’ is a common way of distinguishing one’s experience of early adulthood from that of heterosexual people, especially among white, middle-class lesbians. As a closer reading of the data also suggests, parenthood that arguably happens by accident is often neither unforeseen nor unfortunate, and its currency as a point of reference reveals a powerful cultural narrative. When a wide range of reproductive behaviours are often deemed irresponsible because of their broadly defined timing, describing a pregnancy as an accident obscures responsibility. I argue that, to a certain extent, the discourse of accidental parenthood can serve to prevent moral judgements about reproductive decisions. Consequently, however, the ‘inability’ to have a child by accident makes the prospect of creating a family not only more complicated but also subject to greater scrutiny.

## Introduction

In her 1997 book *Risk and Misfortune: The Social Construction of Accidents*, Judith Green observes that ‘sociology has traditionally largely ignored accidents as a legitimate object of study’ (Green, 1997, p. 7). This lack of attention to accidents is, she argues, intriguing. First, accidents are omnipresent, and even though their existence may seem natural or obvious – and thus not requiring an explanation – their ‘reality’ is socially constructed, and it is vital to grasp how they are understood and managed. Second, the accident is a paradigmatic case of uncertainty and contingency, which are regarded as widespread characteristics of contemporary society, variously described by social theorists as ‘postmodern’ (Bauman, 1991), ‘late modern’ (Giddens, 1991) or as a ‘risk society’ (Beck, 1992). ‘Given the ubiquity of accidents and their importance to understanding the “risk society” in which we live’, Green observes, ‘it is surprising that so few sociologists, even those writing on risk, have addressed them explicitly’ (p. 13).

This article is an attempt to give accidents the overdue sociological attention by exploring their ubiquity in conversations about parenthood, with a focus on how lesbians and gay men, who do not have children but may have them in the future, talk about the possibility of becoming parents. In my study, which explored views about having children in a young generation of lesbian, gay and bisexual people in Britain, lesbians and gay men often commented that, as a same-sex couple, *‘you can’t have a child by accident’*. This was the main way in which they distinguished their experience of early adulthood, in relation to reproduction, from that of heterosexual people, a contrast made especially by (white and largely middle-class) lesbians, with gay men sharing similar views (which, in contrast, were less evident among bisexual people and people of colour). In this article, I argue that ‘accidental parenthood’ is often neither unforeseen nor unfortunate, and its currency as a point of reference reveals a powerful cultural narrative. In a context where a wide range of reproductive decisions are deemed irresponsible because of their broadly defined timing, describing a pregnancy as an accident obscures responsibility. To a certain extent, therefore, the discourse of accidental parenthood may allow people to avoid moral judgements about their reproductive behaviours. Consequently, however, the ‘inability’ to have a child by accident makes the prospect of creating a family not only more complicated but also subject to greater scrutiny.

The article has four main sections. The first section, ‘Accidental pregnancies’, provides an overview of the social science scholarship on reproduction, addressing the phenomenon of ‘unintended pregnancy’ and the notion of ‘intentional parenthood’. The second section, ‘Following accidents’, describes the study this article draws upon and the method used to analyse what in itself was a rather accidental empirical finding. The third section, ‘Narratives of the accident’, tells a story of accidents as they featured in reflections about parenthood among lesbians and gay men interviewed for the study. Finally, the fourth section, ‘Accidentally on purpose’, brings my findings and previous research together to develop an argument that seeks to explain the prevalence of accidental parenthood as a reference point in same-sex narratives of reproduction.

## Accidental pregnancies

A considerable body of research has been dedicated to understanding pregnancies that are ‘unplanned’ or ‘unintended’. Among the many reasons for the scholarly interest in reproductive planning and intention (or lack thereof) – and why, so often, they are not reflected in reproductive behaviours – is the premise that unplanned pregnancies are a problem: they bear the social cost of potentially unwanted children and demand for abortions, as well as associated personal costs (Barrett & Wellings, 2002; Kirkman et al., 2017). The vastness of research in this area is also due to the fact that unintended pregnancy is a complex phenomenon, which is difficult to measure and even define. Studies have consistently shown that it is misguided to fit women’s pregnancies into dichotomous categories such as ‘planned’/‘unplanned’, ‘intended’/‘unintended’ and ‘wanted’/‘unwanted’ (Barrett & Wellings, 2002). This is confirmed by findings from the National Survey of Sexual Attitudes and Lifestyles (Natsal-3), which provides population prevalence estimates of unplanned pregnancy in Britain. In an analysis of Natsal-3 data by Wellings et al. (2013), among women with known pregnancy outcomes, 55% of pregnancies were classified as ‘planned’, 16% as ‘unplanned’ and 29% as ‘ambivalent’, reflecting ‘increasing recognition that many women have mixed feelings about pregnancy’ (p. 1815).

Meanwhile in Australia, Rowe et al. (2016) have estimated that 40% of people who had ever been pregnant or had a pregnant partner had experienced an unintended pregnancy. The authors explain that the pregnancies their findings describe are reported as ‘unintended’ because it is ‘consistent with current usage in the professional literature’ (p. 105). But their report also reveals that, in their survey, pregnancy intention was assessed with a single question, which asked about ‘an *accidental* pregnancy’. As the research team report elsewhere (Kirkman et al., 2017), in the qualitative interviews that were part of the study, the most common term used to describe an unintended pregnancy was ‘accident’ (a term also commonly used by women interviewed in the UK study by Barrett & Wellings, 2002). Rowe et al. (2016) explain further that they had ‘used the lay term “accidental” in the questionnaire because it *implies no judgment* about whether a pregnancy was wanted or not and it can apply to mistimed, unexpected and unplanned pregnancies’ (p. 105, emphasis added).

That ‘accidental pregnancies’ represent a large proportion of parenthood experience links to an increasingly often documented sentiment that there is rarely a ‘right time’ to have children. For example, drawing on interviews with ‘younger and older mothers’ in England, Perrier (2013) notes that, as women try to identify the right time for motherhood, it is difficult for them to ‘synchronise’ different kinds of time: the ‘biographical’ time (having the right partner or the right job) and the ‘psychosocial’ time (feeling emotionally ready for parenthood) are often out of sync with the ‘biological’ time (being physiologically able to conceive). In other words, as women’s biographical and psychosocial ‘resources’ to have children increase, their biological ‘readiness’ for motherhood becomes depleted, which poses challenges to family planning. But social scientists have also pointed out that the idea of there being a ‘right time’ for parenthood is a middle-class notion. Perrier (2013) argues that the cultural narrative of ‘appropriately timed motherhood’ depends on ‘a middle-class life chronology’, marked by specific ‘lifestyle milestones’, such as university education, property ownership and reaching a specific goal in one’s career. These milestones tend to be out of reach for the working class where the labour market prospects offer no incentives to defer parenthood. In fact, it is often having children that provides an impetus for young parents to take up training and employment, which can ultimately improve their social outcomes (Duncan, 2007).

Nevertheless, working-class parents, and young single mothers in particular, are often perceived negatively. In the media, teenage mothers are represented as immoral, promiscuous and over-fertile ‘chav mums’ who pose a threat not only to their children but also to the stability of society (Gillies, 2007; Tyler, 2008). Phoenix (1991) points to two pervasive and conflicting stereotypes of young mothers: the first is that they have children in order to get council housing or welfare benefits and the second is that they become pregnant ‘accidentally’ because they are too ignorant to use contraception. But, as Phoenix points out, the two social constructions of teenage motherhood are logically inconsistent and, in fact, there is no evidence to support either.

As more recent research highlights, women also face social disapproval when they are considered ‘too old’ to have children. Media representations of older mothers portray them as ‘selfish’ for prioritising their careers or ignoring risks associated with conception and pregnancy at an older age (Budds, Locke, & Burr, 2013; Shaw & Giles, 2009). But here, too, empirical evidence paints a different picture of women’s motivations. For instance, recent work on ‘social’ or ‘elective’ egg freezing, which allows women to postpone motherhood while preserving their fertility, shows that far from being career-driven individuals who ‘forget to have children’, women who freeze their eggs do so due to a lack of a partner, as they are unable to find a stable, committed relationship with a man who wants to have children with them (Baldwin, 2018; Inhorn et al., 2018).

Echoing the defence of older women who, prior to childbearing, are keen to create conditions that would enable their children to thrive, a popular argument in support of lesbian and gay parenting is that, because of the necessity of planning for parenthood among same-sex couples, resulting children are always wanted and parents are additionally prepared to take care of their offspring. The discourse of ‘intentional parenthood’ is indeed a common feature in studies of lesbian mothers and gay fathers, which highlight the amount of forward thinking required to create a family as a same-sex couple (Lewin, 2009; Sullivan, 2004). Lesbian couples’ decisions to become parents through donor conception have been described as ‘by necessity’ ‘more deliberative’ (Dalton & Bielby, 2000, p. 59), preceded by ‘a lengthy period of soul-searching’ and informed by ‘much research’, including reading self-help books, watching videos and attending discussion groups (Dunne, 2000, p. 16). Lesbian mothers have been characterised as ‘informed, active consumers of healthcare information and services’ who pursue donor conception in ‘self-actualizing ways’, ‘facing decisions with eyes wide open’ (Mamo, 2007, p. 59). Similar descriptions can be found in the academic literature on gay fathers. Gay men pursuing adoption or surrogacy have been described as approaching parenthood in a way that is ‘highly intentional’ (Goldberg, Downing, & Moyer, 2012, p. 166), with ‘a great deal of planning’ and a sense of ‘heightened responsibility’ (Murphy, 2013, pp. 1114–1115).

It is questionable, however, whether intentionality is fundamental to lesbian motherhood and gay fatherhood, not least because of the high representation of middle-class parents in studies of lesbian-mother and gay-father families. Sociologists such as Gabb (2004) and Taylor (2009) have highlighted that people who come out as lesbian or gay *after* having children belong not only to previous generations but also to specific socio-demographics, and many routes to parenthood for sexual minorities are not accessible to the working class. Most research in this area has been conducted with well-educated and financially comfortable parents in the United States, and social scientists caution against regarding the intentionality of parenthood as universal. For example, Berkowitz and Marsiglio (2007) point out that even though some gay-parent families are formed through conscious planning, many others are not, warning that ‘glowing discourses’ of forethought and detailed planning tend to raise certain families to ‘a romanticized pedestal of responsibility and choice’ (p. 377). Moore (2011) also shows that in some gay communities – notably, among women of colour – having children through step-parenting or heterosexual relationships is more common than pursuing adoption or assisted reproduction. Therefore, the dominant academic narratives of lesbian motherhood and gay fatherhood may obscure the variety of non-heterosexual parenthood experience.

My interviews with lesbians and gay men certainly echoed the discourse of intentional parenthood – partly because they, too, were disproportionately middle-class, but partly also due to the study’s research design, which encouraged interviewees to imagine their futures. The study thus produced data in a form of *prospective* narratives (rather than retrospective accounts of people who were already parents). Similar to the heterosexual participants in the studies by Barrett and Wellings (2002) and Kirkman et al. (2017), interviewees in my study often referred to ‘accidental’ pregnancies. However, they did so in a context of emphasising how their potential experience of parenthood would be *different* from that of their straight peers and parents. In light of the literature reviewed in this section, we may thus ask two related research questions. First, what makes the idea of having a child ‘by accident’ such a strong reference point in talking about parenthood among those who form same-sex relationships? And, second, what does it tell us about broader societal understandings of unintended pregnancy and parenting intentions?

## Following accidents

Data presented in this article come from a qualitative interview study which explored views about parenthood in a young generation of lesbian, gay and bisexual people in Britain. The study examined what men and women in their twenties and early thirties, who had no children, thought about becoming parents in the future. The interviews were conducted in England and Wales between 2012 and 2015.

People who took part in this study had entered their adulthood at a time of increasing possibilities to become parents in a non-heterosexual context. In Britain, different pathways to parenthood opened up for same-sex couples in an exceptionally short period of time. In December 2005, same-sex couples were allowed to jointly adopt (Children and Adoption Act 2002) and the rights of non-biological parents were protected through a new form of relationship recognition (Civil Partnership Act 2004). Over the following few years, it became generally easier to pursue parenthood through assisted conception. For example, the Human Fertilisation and Embryology Act 2008 facilitated access to fertility services for lesbian couples. Changes in law have been accompanied by a more explicit acknowledgement of family diversity by subsequent governments and other institutions, including adoption agencies and fertility clinics, and by an increasing availability of information for prospective parents from sexual minorities.

Interviews were conducted with 23 people, most of whom had been recruited via a dedicated study website. A link to the website was disseminated through multiple channels, including LGBT organisations, LGBT staff networks and Facebook ads. The website described the study as exploring what having and not having children meant to the young generation of non-heterosexual adults in Britain, and targeted people aged 20–35 who *did not* have children. Website visitors could register their interest in being interviewed by completing a short form, which asked a small number of questions, including whether the person wanted to become a parent at some point in the future. The form aimed to select a diverse group of interviewees with respect to their socio-demographic characteristics as well as their views about parenthood.

Of the 23 people interviewed, 12 were men and 11 were women. Interviewees were aged between 23 and 33 years, with a median age of 28. Twenty identified as lesbian or gay and three as bisexual (none identified as transgender). Fifteen were in a same-sex relationship, seven were single and one man was in a relationship with a woman. Nineteen lived in England and four lived in Wales. Twenty-one resided in urban areas and two in rural locations. Twenty were British, one American, one Spanish and one French. Nineteen identified as white, two as black, one as Asian and one as ‘other’. Seventeen had a university degree and six had completed their education at GCSE or A-levels. All but two were employed at the time of our interview and worked in a range of industries. It should be noted that interviewees were predominantly urban, white and middle-class.

Twenty-one interviews were one-to-one and one was with a couple. The interviews, all audio-recorded, lasted between one and three hours. I usually started by asking about interviewees’ initial thoughts upon finding out about the study. With each answer, I prompted them to elaborate on what they had already said. In doing so, I was guided by three broad topic areas, identified in six initial interviews (included in the final analysis): (1) thinking about parenthood (including parenting desires and intentions, or lack thereof), (2) talking about parenthood (including recollections of conversations with partners, family and friends), and (3) attitudes towards different pathways to parenthood (such as adoption, donor conception and surrogacy).

The starting point for this article was an observation that, in my interviews, a particular comment was frequently made unprompted. Interviewees who identified as lesbians, and who were white and largely middle-class, often described parenthood as happening (or not happening) ‘accidentally’, which was usually remarked upon as an aside, without much elaboration. Other interviewees (including men but not bisexual people or people of colour) made similar comments while describing their experiences and possible futures as distinct from those of heterosexual peers. Having been intrigued by a trope that had clearly become a common rhetorical shortcut, I searched through my interview data for mentions of the word ‘accident’, including its variations, and for comments that seemed to convey related sentiments. Finally, I formed a narrative aiming to unpack the various meanings behind references to ‘accidents’ in talking about parenthood.

My analytical approach can therefore be described as ‘following’ or ‘tracing’ a concept – similar to how Ahmed (2006) followed the concept of ‘orientation’ or how, more recently, Epstein and Mamo (2017) traced the concept of ‘sexual health’. I adopt this approach in order to better understand, first, the different meanings of the expression ‘you can’t have a child by accident’ – when used by lesbians and, to a lesser extent, other people who form same-sex relationships; second, why this phrase has seemingly become a common way of distinguishing lesbian motherhood (or non-heterosexual parenthood more broadly) from heterosexual reproduction; and, third, how the frequent use of this idiom changes perceptions of the relationship between reproduction, sexuality, gender and social class. The next section provides an account of how ‘accidents’ featured in my interviews. I refer to interviewees using pseudonyms and, when quoting, I use italics to highlight interviewees’ own emphases.

## Narratives of the accident

The key aim of this study was to explore how people who form same-sex relationships approach the topic of parenthood when they think about the future. As I discuss elsewhere (Pralat, 2016, 2018), people who took part in the interviews articulated their feelings about the prospect of having children by drawing on examples of parenthood they were personally most familiar with – that is, parenthood of their own (heterosexual) parents and peers. In doing so, interviewees often framed their comments in terms of similarities or differences between heterosexual and non-heterosexual experiences of, and expectations about, reproduction. On the one hand, it was striking how frequently and spontaneously they used language that could be described as heteronormative. The use of phrases such as ‘at this stage of my life’, ‘natural progression’ and ‘biological clock’ seemed somewhat taken for granted, and interviewees rarely remarked on their lexicon’s association with conventional understandings of the life course or the nuclear family. On the other hand, when contextualising their thoughts about parenthood, interviewees usually recounted *how* their own parents and straight friends had welcomed children into their lives – and it was here where contrasts were drawn. Most commonly, those transitions to parenthood were reported to have happened without much, if any, preparation and they were often described as unplanned or unintended. In this context, interviewees positioned themselves as different from the heterosexual majority, despite the fact that, in so many other ways, there was nothing distinctly ‘gay’ in how they thought about family and personal life.

One of the most often occurring comments in my interviews was an observation that becoming a lesbian or gay parent did not happen ‘by accident’. Ruth, a 24-year-old lesbian in a long-term relationship, reflected: ‘You know, family planning in a gay couple is harder – you can’t accidentally get pregnant as a gay couple.’ Having been together for six years and cohabited most of this time, Ruth and her partner felt almost ready to welcome children into their home, so far occupied by the two women and their four pets. Partly because the couple did not know any men who could act as their sperm donors, they thought about using a sperm bank and inseminating in a clinic. Illustrating what exactly made family planning more difficult for same-sex partners, Ruth compared herself with her heterosexual sister:

If my sister wanted to have a baby, she could just stop taking the pill. That’s all she’d have to do. She has sex with her boyfriend anyway. It’s not like they have to change their behaviour in any way – she just stops taking the pill. For me, I have to go through six months of medical appointments, and saving for two years, and keeping a diary of what days I’m on my period, and peeing on a stick to tell when I’m ovulating, and all of this stuff that straight couples don’t necessarily *have* to do. I *have* to do it that way.

By describing in detail what most straight couples do not have to do in order to become parents, Ruth portrayed heterosexual reproduction as effortless. While some interviewees referred to cases of infertility among straight people they knew, in the majority of references to heterosexual individuals parenthood had happened ‘simply’ through having sex. Scott, a gay man aged 29, shared Ruth’s sentiments: ‘It’s so easy for a man and a woman – you pop upstairs, come back down, [wait] nine months, and a baby’s born. Where for us it’s so difficult.’ Other interviewees noted that, in straight partnerships, ‘you wouldn’t even have to think’ about how to have children – ‘you’d just do it’. Louis, a 24-year-old gay man, pointed out the financial dimension of becoming a parent:

You know, if you’re a heterosexual couple with functioning genitals, you can be as poor as you want and you will have kids. How you raise them and whether or not the state supports that and all that stuff is a whole other issue, but the process of acquiring the children is free.

Money, finances and, by extension, social class played a key role in these same-sex narratives of family planning. When interviewees talked about clinical insemination or surrogacy, the costs of becoming a parent were often illuminated – both men and women saw the pursuit of parenthood as inevitably involving substantial expense. But the issue of resources was highlighted from a different perspective when interviewees reflected on the socioeconomic disadvantage associated with ‘accidental pregnancy’. Here, the spontaneous nature of heterosexual reproduction, rather than evoking jealousy, brought a kind of relief about one’s own circumstances. This was especially the case among those interviewees whose social circles included single parents and, more specifically, women parenting alone.

Interestingly, interviewees from working-class backgrounds, or ones whose peers struggled financially to raise children on their own, sometimes talked about homosexuality as if it was giving them the ability to exercise reproductive control. Seeing privilege in being a gay man, Scott observed: ‘A lot of us have got female friends who are single mothers and [we] see how hard it is. We’ve got the choice not to have it, you see.’ In some interviews, being lesbian or gay shaped the process of imagining not only the future but also an alternative, what-if present. Thom, a gay man aged 23, shared an insightful reflection during our interview:

Thom: If I could reproduce myself, if I could get pregnant, I probably would have had a child by now, I think.

Robert: Why do you think so?

Thom: I don’t know. Because . . . Obviously, I think it would just be easy. And I think because a lot of my friends got pregnant . . . I think there’s about eight girls from my year at school – all pregnant around the same time. I think it was just a sort of thing to do. But, yeah, I think if I could have a child naturally . . . I don’t know, I think I probably would [have had children by now].

Compared with the earlier quotation from my interview with Ruth, Thom’s account gives a different perspective on the ‘easiness’ of heterosexual reproduction – here, advantage and procreation are related in a different way. Whereas the heterosexuality of Ruth’s sister retained agency and conscious decision making (‘if my sister wanted to have a baby, she could just stop taking the pill’), these elements appear missing in Thom’s description of his former classmates. Based on his account, there was little reflection over becoming a parent among the young women he knew from school – ‘it was just a sort of thing to do’. Thom doubted that he would have avoided having children at a young age himself if he was straight, highlighting how reproduction can be outside of one’s control. Tellingly, while putting himself in ‘straight shoes’, like Scott earlier, he identified with his female peers rather than the fathers of the women’s children. Heterosexual men were absent from interviewees’ narratives, which illuminates a complex relationship between reproduction, sexuality, gender and social class.

As Thom’s comments make clear, the inability to unintentionally ‘reproduce oneself’ in gay sex has implications beyond ‘family planning’ – to a large degree, it can shape people’s approach to life as a whole. One potential advantage of the ‘impossibility’ of accidental parenthood is a greater sense of control over the future. This sense of control, in turn, affects attitudes towards sex, which becomes dissociated from some of its reproductive connotations. For example, Lauren, a 30-year-old lesbian uninterested in becoming a parent, did not envy her heterosexual peers: ‘I don’t know *how* my straight friends handle the *fear* of that, like, you know, every month thinking, “Oh crap, I’m late”, etcetera, etcetera. I just can’t even *imagine* that fear!’ While having a late period could be exciting for Ruth, the prospect of delayed menstruation filled Lauren with terror. We can see how the relationship between reproduction and sexuality intersects not only with gender and social class, but also with parenting desire (or lack thereof), making life more difficult for some while playing in favour of others.

There are thus both costs and benefits in the inability to have children ‘by accident’, and most interviewees gave nuanced accounts, balancing advantages and disadvantages of their situation rather than positioning themselves as simply unlucky or fortunate. For instance, Becky, a lesbian aged 25, recalled her coming out – a transition that made her consider issues she had not pondered before:

When I realised I was gay, particularly as I’d been in a heterosexual relationship for three years, obviously I had some questions as to how [I would become a parent]. But, again, I never really waived. I just thought to myself that it’s gonna be different, it’s gonna be really different, and it’s gonna – it’s not going to happen by accident, let’s face it. [*laughs*] So in lots of ways I’m quite glad because it’ll be the most wanted and planned child possible. But, at the same time, it’s kind of sad – no, it’s not sad – I find it upsetting that I can’t have, I can’t just, like, stop using protection, say it will happen, and take the worry off it. *That’s* what worries me – that it’s gonna have to be really planned. It’s gonna have to happen some way that is really, really planned, there’s a lot of stress and there’s a lot of, like, focus on more moments. *That’s* what worries me, going forward.

The improbability of falling pregnant by chance, as it were, had pros and cons for Becky. It made her convinced that she would have ‘the most wanted and planned child possible’. But she also got frustrated that she could not simply stop using contraceptives. In the above quotation, there is a tension between a desirable intentionality, enabling prospective parents to put things in place before their child is conceived, and a deliberateness that becomes a burden because of its intensity. While it is favourable for children to be planned, it can be worrying when they have to be ‘really, really planned’.

Similar to Becky, Gavin, a 25-year-old gay man, saw both sides of the coin in the intentional character of non-heterosexual reproduction. Hoping to have a child via surrogacy with his partner, he also expressed concerns about planning. The couple differed in the extent to which they had been ‘exposed’ to pregnancies and births in their respective families, which made them approach the prospect of parenthood in contrasting ways. Gavin seemed less concerned than his partner about thinking ahead:

I think, if we were a heterosexual couple, it would never warrant this much discussion or this much planning. For instance, [our friend], she’s just got pregnant, and they *dealt* with it, that’s it, you know. So, yeah, I think [my partner] kind of sets himself up to fail in a sense, where he kind of just has to cover every eventuality, every bloody topic. I think, I suppose it is necessary, because I suppose it develops your awareness and makes you prepared. But then is it *really* that necessary to go through *every single* bloody detail?

With varying approaches to parenthood, Gavin and his partner needed to negotiate the extent to which they wanted to plan while pursuing their desire to become parents. As Gavin noted, there was a risk of setting oneself up for failure if the couple got carried away with looking forward. It seems that becoming a parent required reducing the temptation to ‘cover every eventuality’ by accepting that sometimes you just ‘deal with it’.

Sally, a lesbian aged 31 with a partner of seven years, told me about parents among her friends, which led her to observe that lack of planning for parenthood was common: ‘One of the things about my friends who’ve had children is that a *large* percentage of them have been accidents. So it’s not like they’ve made the decision to have children. Obviously that’s not going to happen with us, so it’s . . . it’s a little bit harder.’ Comparisons *across* generations also showed that not only pregnancies that were unplanned but also pregnancies that were (initially, at least) unwanted were not unusual. Lauren, for instance, recalled her childhood:

My mum told my sister and me quite regularly [when we were] growing up that she didn’t actually want children – it was just an accident and they made the best of it. It wasn’t quite as heartless as it sounds! [*laughs*] My mum’s really good, she’s really loving and really fabulous. But I think it was one of those, ‘Oh . . . well, better make the best of this!’

Even though, as Lauren remarks, the idea of a mother telling her children that they were not wanted may sound ‘heartless’, this example shows that ‘accidental parents’ are not necessarily worse than ‘intentional parents’ – they can be just as ‘good’ and just as ‘loving’, and the resulting parent–child relationship can be equally harmonious. In fact, the accidental nature of parenthood may also benefit the relationship between partners, as Lauren explained later in our interview:

[With] straight couples, ’cause you can have kids by accident, you know, things happen and you make the best of it, and stuff like that. But with two women . . . it’s quite an effort. And it’s got to be a very kind of decisive, *definite* thing to decide. ‘We’re gonna have children, we’re gonna try and have children.’ I’ve got some friends who are going through this at the moment, and it’s quite a slog.

Here we have an account that runs counter to the narrative of accidental pregnancy as likely to result in relationship breakdown due to limited communication about parenthood between partners prior to conception. Instead, accepting that ‘things happen’ and making ‘the best of it’ can be less of a challenge for partners than the effort required when reproductive decisions are approached prospectively in a conscious manner. The ‘definite’ character of intentional parenthood can make the process of creating a family become ‘quite a slog’, with potentially negative effects on the dynamics within the couple, who, as comments from Becky and Gavin have already shown, need to engage in difficult conversations. If a pregnancy happens ‘by accident’, partners can avoid facing these difficulties.

It is not only communication between romantic partners that can be challenged when parenthood needs to be planned; it is also communication outside of the couple. Sally imagined a situation when she would need to announce a pregnancy in her workplace: ‘It’s not like you can turn around to your boss and go, “It was an accident!” You know, *obviously* it wasn’t an accident! [*laughs*] But to what extent do you – yeah . . . That could potentially be a very difficult conversation.’ Even if employers are increasingly accepting of family diversity, we can see how (openly out) lesbians cannot follow a script that is seemingly available to other women. Describing one’s pregnancy as an accident might serve as a form of protection for one’s career, even if the accident was, in fact, ‘planned’. Such ‘pretended accidents’ may, to some extent, prevent judgements about women’s commitment to work. They cannot, however, protect women whose decisions to have children at a given time are clearly deliberate.

Although often unpleasant, conversations that bring the prospect of planning for parenthood to the fore can also be constructive for intended parents. Having been introduced to the personal lives of many same-sex couples, it became apparent to me that the inevitability of planning facilitated dialogue for those who did want to create a family, even if the conversations were not always easy. Vicky, a lesbian aged 28, provided further insights into the additional efforts related to the kind of parenthood that can only happen intentionally:

[My partner and I] did have some sort of pre-parenting counselling, just to kind of make sure that this was something that we wanted to do. . . . And I think when you are a lesbian couple, or even a gay couple, because it’s not so natural and you can’t *just* get pregnant by accident, you do kind of overthink things, and you do think long and hard about it. And it probably was a bit excessive to go and have counselling – you know, it wasn’t an extra step that straight couples necessarily take – but it was very beneficial, very useful in terms of helping us to communicate about having children.

Vicky and her partner’s decision to have ‘pre-parenting counselling’ suggests that the couple felt like they needed to go the extra mile to reassure themselves that they were ready for parenthood. However, as the earlier quotations also show, the principle that plans to create a family have to be well thought out includes ensuring that one does not, in Vicky’s words, ‘overthink things’. Therefore, the most preferable approach to reproductive decision making emerging from the interviews is not only to be reflective but also to reflect in moderation. Any ‘excessiveness’ of planning appears beneficial to the extent it improves communication between partners so that they can embark on their journey to parenthood in a balanced and resilient way. But why is positioning oneself vis-a-vis people who can ‘*just* get pregnant by accident’ so common in articulating one’s approach to parenthood? Bringing together my data and existing scholarship, I will now seek to offer an explanation.

## Accidentally on purpose

By tracing the concept of ‘accidental parenthood’ in my data, I have attempted to draw attention to its ostensibly central role in positioning sexual-minority parenthood as different from heterosexual reproduction, amid a myriad of similarities. Having presented how interviewees, especially lesbians, talked about the inability to have children ‘accidentally’, I will now discuss my findings in dialogue with existing scholarship in order to understand what exactly the insights described in the previous section tell us about the changing perceptions of the relationship between reproduction, sexuality and various axes of cultural difference, in particular, gender and social class.

I begin my argument by suggesting that, in my interviews, the frequency of references to spontaneous conception, including accidental parenthood, highlights the centrality of ease and effortlessness in cultural understandings of heterosexual reproduction and its normativity. Lesbians, gay men and same-sex couples – as well as straight people who, for a variety of reasons, are unable to conceive despite wanting to – can envy (fertile) heterosexuals the ‘ease’ of reproduction, not only because parenthood that happens ‘simply’ through having sex makes having babies cheaper and more achievable, but also because it is closely linked to the ideal of the romantic love. ‘Forgetting’ to use contraception while getting ‘caught in the moment’ is a common experience among heterosexual people (Brown & Guthrie, 2010) and, apart from having potentially negative consequences for individuals involved, it also has positive connotations. Romance plays a prominent role in the cultural narrative of ‘natural’ reproduction, where partners (and future parents) are intimately connected. As Mamo (2007) observes, this narrative ‘moves into the lesbian-insemination script’ (p. 146): stories of donor insemination performed at home, as opposed to a fertility clinic, often include romantic elements, featuring music, candles and even sexual intimacy, and lesbian couples who do end up using a fertility clinic, after unsuccessfully trying to conceive at home, frequently recall their previous attempts at self-insemination with nostalgic references to fun, thrill and excitement. In my study, comments from those wanting to become parents in the future, like Ruth, Scott and Becky, echo the frustration expressed by lesbian mothers about the, from their perspective, unfortunate separation of reproduction from sex.

But recognising the heteronormativity of reproduction does not in itself explain the currency of accidental parenthood as a trope. I would argue that what is demarcated by interviewees in my study as beyond their reach is not only reproduction that is easy, effortless and spontaneous; it is also – precisely by being described as happening by accident – reproduction that is immune from scrutiny. Green (1997) notes that making ‘claims to the accidental’ disclaims responsibility. In other words, people cannot be held responsible if what they did was an accident – if it was not done on purpose. Luker (1999) makes a similar observation writing specifically about pregnancy. ‘Becoming “accidentally” pregnant’, she argues, ‘permits people to duck the onerous responsibility of having to *decide* whether to enter into parenthood’ (p. 249, emphasis in original). Existing empirical evidence highlights that heterosexual people often describe unplanned or unintended pregnancies as accidents (Barrett & Wellings, 2002; Kirkman et al., 2017). My data additionally illuminate the prominent role of this description in lesbian and gay people’s perceptions of heterosexual reproduction, which confirms that claiming to have had a child by accident, or the ability to make this claim, can be appealing. But why would becoming pregnant ‘on purpose’ be so prone to being perceived as irresponsible?

Based on my data as well as sociological work on heterosexual parenthood, I would argue that accidental parenthood allows people to avoid the responsibility of having to decide not so much *whether* to enter into parenthood, as Luker suggests, but *when* to do so. Early in the article, we saw that it is often difficult for people, and for women in particular, to identify a ‘right time’ to become parents (Perrier, 2013). If there is rarely, if ever, a right time to have a child, it follows that reproductive decisions are often regarded as ‘mistimed’. As such, these decisions are likely to attract moral judgements, whether they relate to parental age, work situation or relationship status. Therefore, parents, and mothers especially, are often described as irresponsible because of the *timing* of their parenthood: for having children too young or too old, at a critical moment in one’s career, or with an insufficiently long-term partner (if they have a partner at all). Considering the ‘high risk’ of being judged as irresponsible for having a child at a wrong time, claiming accidental parenthood can prevent judgements, because the ‘wrong time’ was not deliberately chosen. Indeed, if judgements can be avoided in this way, describing one’s parenthood as accidental – even when what it actually means is ‘accidental on purpose’ – seems like a responsible thing to do in itself.

It is important to emphasise that comments about accidents made by interviewees in my study, as they described the parenthood experience of straight people, did not refer to becoming pregnant accidentally due to ignorance about contraception – a stereotype of young mothers discussed by Phoenix (1991). Instead, interviewees’ comments alluded to rather benevolent accidental pregnancies that happen within a context of a stable relationship where partners usually, though not always, want to have children anyway, even if it is not exactly at the time it ends up happening. We can understand such pregnancies as ‘accidents waiting to happen’ where resulting parenthood is more likely to be a ‘hap’ than a ‘mishap’, even if the way in which it is talked about suggests otherwise.

Approaching the accidental from this perspective sheds new light on the understanding of the relationship between the intentionality of parenthood, reproductive responsibility and social class. As discussed early in the article, the discourse of reproductive responsibility is often invoked in a way that stigmatises working-class women. Emphasising the intentionality of sexual-minority parenthood, by claiming the righteousness of one marginalised group at the cost of another, may contribute to this stigmatisation – even if doing so is not intentional. But there is little evidence in my data of pejorative discourses among middle-class lesbians and gay men towards working-class heterosexuals. Comments about unplanned parenthood did not reference ‘reckless behaviours’, ‘promiscuous lifestyles’ or other characteristics stereotypically attributed to the working class. To be sure, non-heterosexual parenthood *was* perceived as necessarily intentional, but its compulsory deliberateness was not seen as a virtue in itself. On the contrary, it was precisely the purposeful nature of gay parenthood that seemed to raise concerns about irresponsibility. As such, rather than reflecting judgements about women who become mothers ‘too young’, my findings echo criticisms of women who have children ‘too old’ and the increasing recognition of the moral scrutiny of middle-class parenthood, manifested in tensions between the importance of planning ahead and the impossibility of being fully prepared for having children.

In this context, where accusations of reproductive irresponsibility encompass both the working class and the middle class, I would argue that having children ‘accidentally on purpose’ has become a powerful, if not dominant, narrative. When for the majority of the middle class achieving certain lifestyle milestones, such as job security and home ownership, is delayed (sometimes indefinitely), and women are expected to both become mothers and sustain successful careers, parenthood through ‘pretended’ or ‘planned’ accidents may be a new kind of norm. Rather than an exception, such ‘accidental’ parenthood has potentially become a rule because of its ability to prevent judgements about whether becoming a parent at a given time is compatible with the stage of one’s career, the balance of one’s bank account or the state of one’s relationship. Combined with the heteronormative ideal of spontaneous conception discussed earlier, it makes sense that ‘having a child by accident’ can be peculiarly appealing. However, this accidentally-on-purpose parenthood is not accessible to everyone. Any protection from scrutiny that claiming accidental parenthood can provide is less likely to benefit the working class than the middle class and, for different reasons, it is out of reach for lesbians and gay men. Assisted reproductive technologies and the increasing social acceptance of family diversity may have facilitated a wider range of parenthood possibilities, including creating a family as a same-sex couple. But as different family forms become more normalised, the privileging of ‘natural’ reproduction seems to manifest itself in more implicit ways.

## Conclusion

This article has aimed to demonstrate that, though counterintuitive, it is a worthwhile endeavour to look for the meaning of unintended pregnancy among people who, in their own view, cannot experience it. Responding to the call by Green (1997), I have taken accidents more seriously as an object of study by examining the role they play in the everyday management of risk and uncertainty. More specifically, I have shown that views about having children among people who form same-sex relationships are highly influenced by the phenomenon of ‘accidental parenthood’, even if its existence is mainly discursive. My findings shed new light on the understanding of lesbian motherhood and gay fatherhood as intentional by illuminating a more complex relationship between intentionality and privilege. I have suggested that the notion of having children ‘accidentally on purpose’, in its ability to disclaim reproductive responsibility, is a powerful cultural narrative, especially among the middle class, and I would encourage researchers to further examine the validity of this argument.

As this article draws on small-scale qualitative research, other arguments I have made about reproduction, sexuality, gender and social class should be viewed within the context of the study’s limitations, and these arguments too are worth examining further. For example, even though I cannot draw definite conclusions based on my data, it is noteworthy that some gay men in my study, when using heterosexual reproduction as a point of reference for their own thinking about parenthood, compared themselves with female peers, not with straight males. Likewise, it is notable that none of the interviewees who identified as bisexual and none of those who were black or Asian used the term ‘accident’ at any point during the interview, nor did they emphasise the contrast between heterosexual and sexual-minority parenthood by referring to unintended pregnancy. These indicative findings highlight the importance of thinking about reproduction in an intersectional way, and future studies should further attend to how sexual and ethnic identities, as well as gender and class, shape people’s perceptions of parenthood.
